# The MYB transcription factor PbMYB12b positively regulates flavonol biosynthesis in pear fruit

**DOI:** 10.1186/s12870-019-1687-0

**Published:** 2019-02-21

**Authors:** Rui Zhai, Yingxiao Zhao, Meng Wu, Jie Yang, Xieyu Li, Hanting Liu, Ting Wu, Fangfang Liang, Chengquan Yang, Zhigang Wang, Fengwang Ma, Lingfei Xu

**Affiliations:** 1 College of Horticulture, Northwest A&F University, Taicheng Road NO.3, Yangling, Shaanxi Province China; 2 State Key Laboratory of Crop Stress Biology for Arid Areas, Northwest A&F University, Taicheng Road NO.3, Yangling, Shaanxi Province China

**Keywords:** Flavonol, MYB12, Fruit, Pear

## Abstract

**Background:**

As a class of natural antioxidants in plants, fruit flavonol metabolites are beneficial to human health. However, the regulatory networks for flavonol biosynthesis in most fruits are largely unknown. Previously, we reported a spontaneous pear bud sport ‘Red Zaosu’ (*Pyrus bretschneideri* Rehd.) with a high flavonoid content in its fruit. The identification of the flavonol biosynthetic regulatory network in this mutant pear fruit is crucial for elucidating the flavonol biosynthetic mechanism in fruit.

**Results:**

Here, we demonstrated the *PbMYB12b* positively regulated flavonols biosynthesis in ‘Red Zaosu’ fruit. Initially, we investigated the accumulation patterns of four major quercetin glycosides and two major isorhamnetin glycosides in the fruit of ‘Red Zaosu’ and its wild-type ‘Zaosu’. A PRODUCTION OF FLAVONOL GLYCOSIDES (PFG)-type MYB transcription factor *PbMYB12b* was also screened for because of its correlation with flavonol accumulation in pear fruit. The biofunction of *PbMYB12b* was verified by transient overexpression and RNAi assays in pear fruit and young leaves. Overexpression of *PbMYB12b* enhanced the biosynthesis of quercetin glycosides and isorhamnetin glycosides by positively regulating a general flavonoids biosynthesis gene *PbCHSb* and a flavonol biosynthesis gene *PbFLS*. This finding was also supported by dual-luciferase transient expression assay and transient β-glucuronidase (GUS) reporter assay.

**Conclusions:**

Our study indicated that *PbMYB12b* positively regulated flavonol biosynthesis, including four major quercetin glycosides and two major isorhamnetin glycosides, by promoting the expression of *PbCHSb* and *PbFLS* in pear fruit.

**Electronic supplementary material:**

The online version of this article (10.1186/s12870-019-1687-0) contains supplementary material, which is available to authorized users.

## Background

Flavonoids represent a large group of secondary metabolites in plants that generally have a C6-C3-C6 carbon skeleton [1]. Anthocyanins, flavonols, flavanols and flavanones are common flavonoid compounds found in most fleshy fruits [2–5]. There are various flavonol biofunctions in plants. Antioxidant capacity is a common feature of flavonols and most other flavonoid compounds. In addition, flavonols also have some unique biofunctions. Flavonol derivatives, especially quercetins and kaempferols, have the capacity to inhibit auxin transport [6]. Moreover, flavonols may be involved in the fertilization processes of some higher plants [7, 8].

**Fig. 1:**
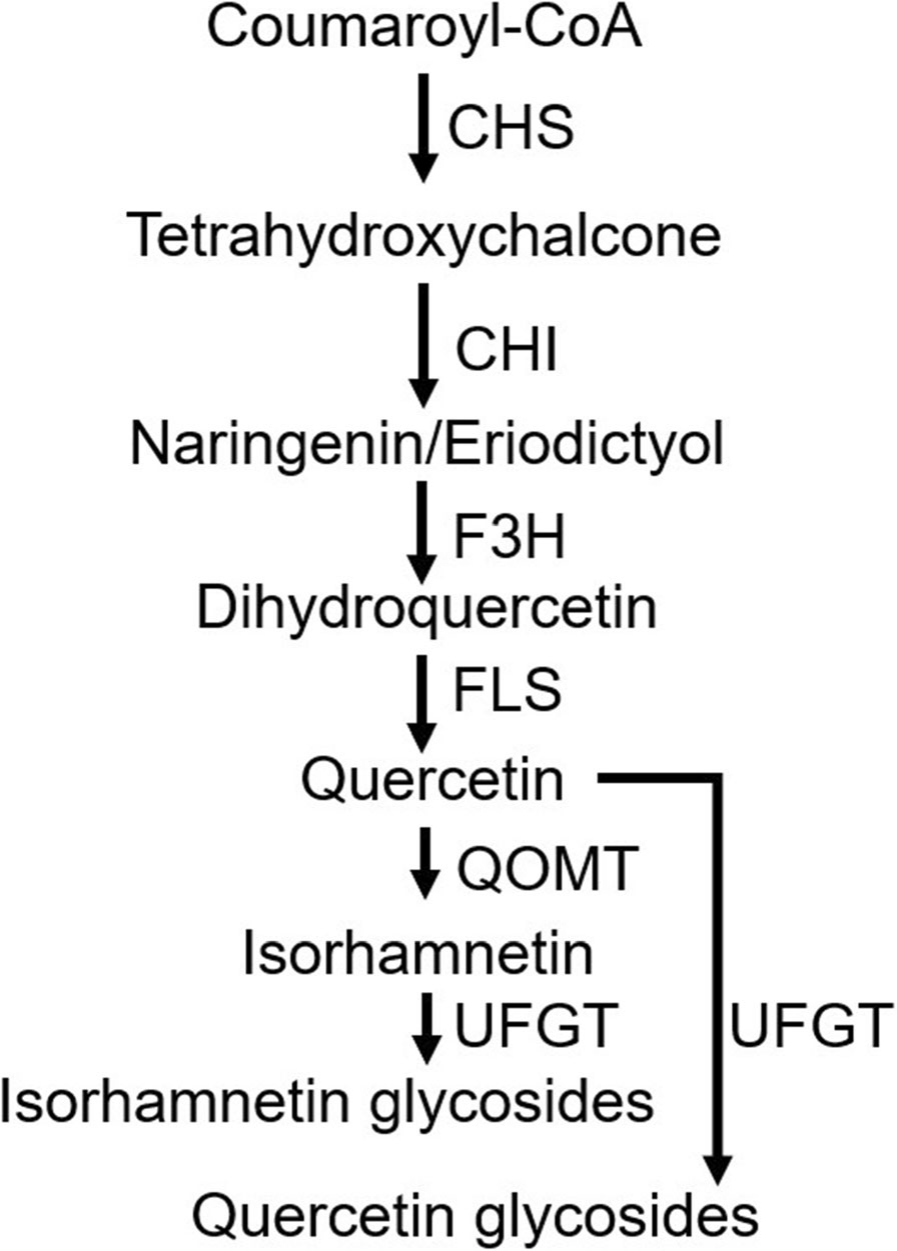
Flavonol glycoside biosynthetic pathway in pear fruit

The biosynthetic pathways of most flavonoid compounds have been verified. The flavonoid biosynthetic pathway is part of the phenylpropanoid pathway. Specifically, phenylalanine ammonia lyase, cinnamate 4-hydroxylase and 4-coumarate: CoA ligase are the biosynthetic enzymes in the general phenylpropanoid pathway. Chalcone synthase (CHS), chalcone isomerase (CHI), and flavanone 3-hydroxylase are the common biosynthetic enzymes of most flavonoid compounds. Flavonol synthase (FLS), UDP-glucose: flavonoid 3-glucosyltransferase (UFGT) and quercetin 3-O-methyltransferase (QOMT) are specifically involved in the biosynthesis of flavonol compounds (Fig. 1) [3, 9].

The flavonoid biosynthetic genes, which encode these enzymes, are transcriptionally regulated by MYB family transcription factors (TFs). Three subgroups among the MYB family were identified as typical activators of the anthocyanin, flavanol and flavonol pathways, respectively [10–12]. To be more specific, PRODUCTION OF ANTHOCYANIN PIGMENT (PAP)-type MYBs, including *PAP1(AtMYB75)*, *PAP2(AtMYB90)*, *AtMYB113* and *AtMYB114* in *Arabidopsis*, *MYB10* in apple (*Malus × domestica*), and *MYB10* and *MYB10b* in pear (*Pyrus bretschneideri* Rehd*.*), were identified as positive regulators of the anthocyanin pathway [10, 11, 14, 15]. TRANSPARENT TESTA 2 (TT2)-type genes, including *TT2* in *Arabidopsis*, *PbMYB9* in pear and *MdMYB9* in apple, were identified as positive regulators of the flavanol pathway [12, 15–17]. PRODUCTION OF FLAVONOL GLYCOSIDES (PFG)-type MYB TFs were first identified as positive regulators of the flavonol pathway in *Arabidopsis* [18]. These three different types of MYB TFs, PAP, TT2 and PFG, specifically activate the genes encoding the flavonoid biosynthetic enzymes involved in the anthocyanin, flavanol and flavonol pathways, respectively. PAP-type MYB TFs transcriptionally activate the genes encoding leucoanthocyanidin dioxygenase, dihydroflavonol 4-reductase and UFGT [10, 12]. TT2-type MYB TFs transcriptionally activate the genes encoding leucoanthocyanidin reductase and anthocyanidin reductase [16]. PFG-type MYB TFs transcriptionally activate the genes encoding CHS, CHI and FLS [13, 18].

Fleshy fruits are the primary dietary sources of flavonols. Several PFG-type MYB TFs were screened from some fleshy fruits. In grapevine (*Vitis vinifera*), *VvMYBF1* was verified in positively regulating flavonol biosynthesis by activating CHS and FLS [19]. *MdMYB22* was proposed as a positive regulator of the flavonol pathway by inducing the expression of *MdFLS* in apple [20]. The Chinese pear ‘Red Zaosu’ originated as a spontaneous mutation from ‘Zaosu’ (*P. bretschneideri* Rehd*.*). The accumulation levels of anthocyanins and flavonols are much greater in ‘Red Zaosu’ fruit than in ‘Zaosu’ fruit [9]. Previously, we verified that *PbMYB10b* and *PbMYB9* are positive regulators of the anthocyanin and flavanol pathways, repectively [15]. However, the flavonol biosynthetic regulatory network in pear fruit is still poorly understood.

In this study, we initially screened for candidate genes that may be involved in flavonol biosynthesis in ‘Red Zaosu’ fruit using a comparative transcriptome analysis. The general flavonoids biosynthesis genes *PbCHSa* and *PbCHSb*, the flavonol biosynthesis genes *PbFLS* and *PbUFGT*, and one PFG-type MYB TF, *PbMYB12b*, were used. *PbCHSb*, *PbFLS* and *PbMYB12b* were further investigated because of their high correlations with flavonol glycoside accumulation patterns in ‘Red Zaosu’ fruit. *PbMYB12b* activated the expressions of *PbCHSb* and *PbFLS*, resulting in flavonol glycoside accumulations in ‘Red Zaosu’ fruit. Thus, a *PbMYB12b*-regulated flavonol biosynthetic pattern in pear fruit was revealed.

## Results

### PbMYB12b is a potential regulator of the flavonol biosynthetic pathway in pear fruit

**Fig. 2:**
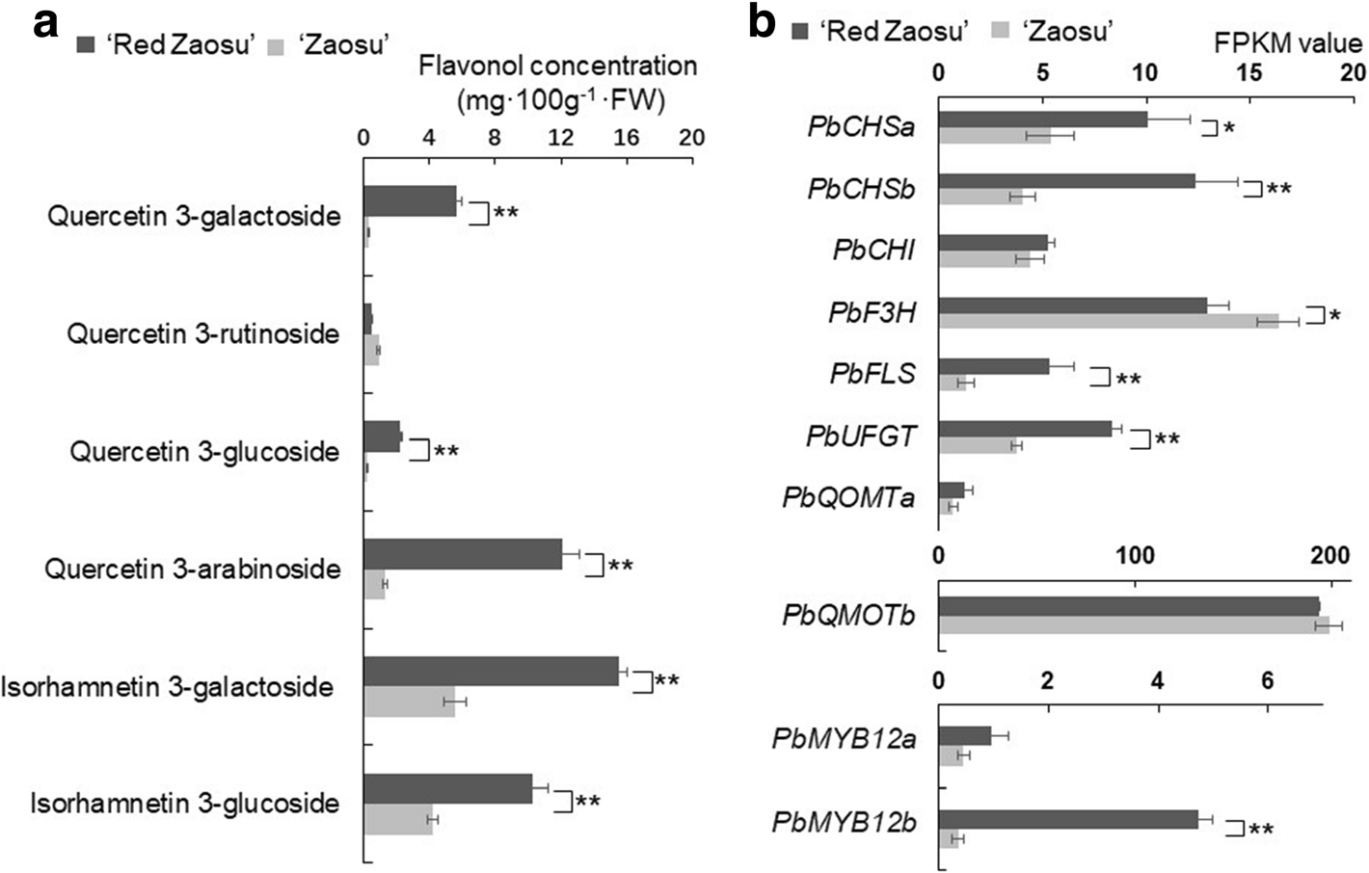
Flavonol glycoside concentrations and the expression patterns of related genes in pear fruit. **a** Flavonol glycoside concentrations in skin of ‘Red Zaosu’ and ‘Zaosu’ fruit during the fruit developing period (DP). Data are the means ± SDs of five biological replicates. Asterisks indicate significant differences between ‘Red Zaosu’ and ‘Zaosu’ as assessed by Student’s t test: **P < 0.01. **b** The expression patterns of flavonol biosynthetic genes in ‘Red Zaosu’ and ‘Zaosu’ fruit during the DP. Data are the means ± SDs of three biological replicates. Asterisks indicate significant differences between ‘Red Zaosu’ and ‘Zaosu’ as assessed by Student’s t test: ^*^P < 0.05, ^**^P < 0.01

To investigate the flavonol biosynthetic patterns in the leaves and fruit of ‘Red Zaosu’ and its wild-type ‘Zaosu’, the concentrations of flavonol compounds in their tissue cultured young leaves (three-day-old) and their fruit skin at the fruit developing period (DP) were initially investigated. Quercetin 3-galactoside, quercetin 3-glucoside, quercetin 3-rutinoside, quercetin 3-arabinoside, isorhamnetin 3-galactoside and isorhamnetin 3-glucoside were the major flavonol glycosides found in the fruit skins of ‘Red Zaosu’ and ‘Zaosu’ (Fig. 2a). Only four major flavonols compounds including quercetin 3-galactoside, quercetin 3-glucoside, isorhamnetin 3-galactoside and isorhamnetin 3-glucoside were identified in pear leaves (Additional file 1: Figure S2a). We compared the concentrations of these flavonol compounds between ‘Red Zaosu’ and ‘Zaosu’. The concentrations of most flavonol glycosides were significantly greater in ‘Red Zaosu’ than in ‘Zaosu’ (Fig. 2a, Additional file 1: Figure S2a).

Furthermore, the expression patterns of flavonol biosynthetic genes were screened from a comparative transcriptome analysis between the DP fruit skins of ‘Red Zaosu’ and ‘Zaosu’ (Fig. 2b, Additional file 1: Table S1). Several flavonol biosynthetic genes including *PbCHSa, PbCHSb, PbFLS* and *PbUFGT* were differentially expressed between ‘Red Zaosu’ and ‘Zaosu’. Consistent with most flavonol glycosides accumulation patterns, the expression levels of *PbCHSa*, *PbCHSb*, *PbFLS* and *PbUFGT* in ‘Red Zaosu’ fruit and leaves were greater than those in ‘Zaosu’ (Fig. 2b, Additional file 1: Figure S2b). Moreover, two PFG-type MYB TFs, designated *PbMYB12a* and *PbMYB12b*, were expressed in the transcriptome of the DP fruit skins of ‘Red Zaosu’ and ‘Zaosu’ (Additional file 1: Table S1). The expression level of *PbMYB12b* was significantly greater in ‘Red Zaosu’ fruit and leaves (Fig. 2b, Additional file 1: Figure S2b). The expression of *PbMYB12a* in the two cultivars did not show significant difference neither in fruit nor in leaves (Fig. 2b, Additional file 1: Figure S2b). Thus, *PbMYB12b* was investigated further as a potential regulator of flavonol biosynthesis.

### Identification and molecular characterization of PbMYB12b

**Fig. 3:**
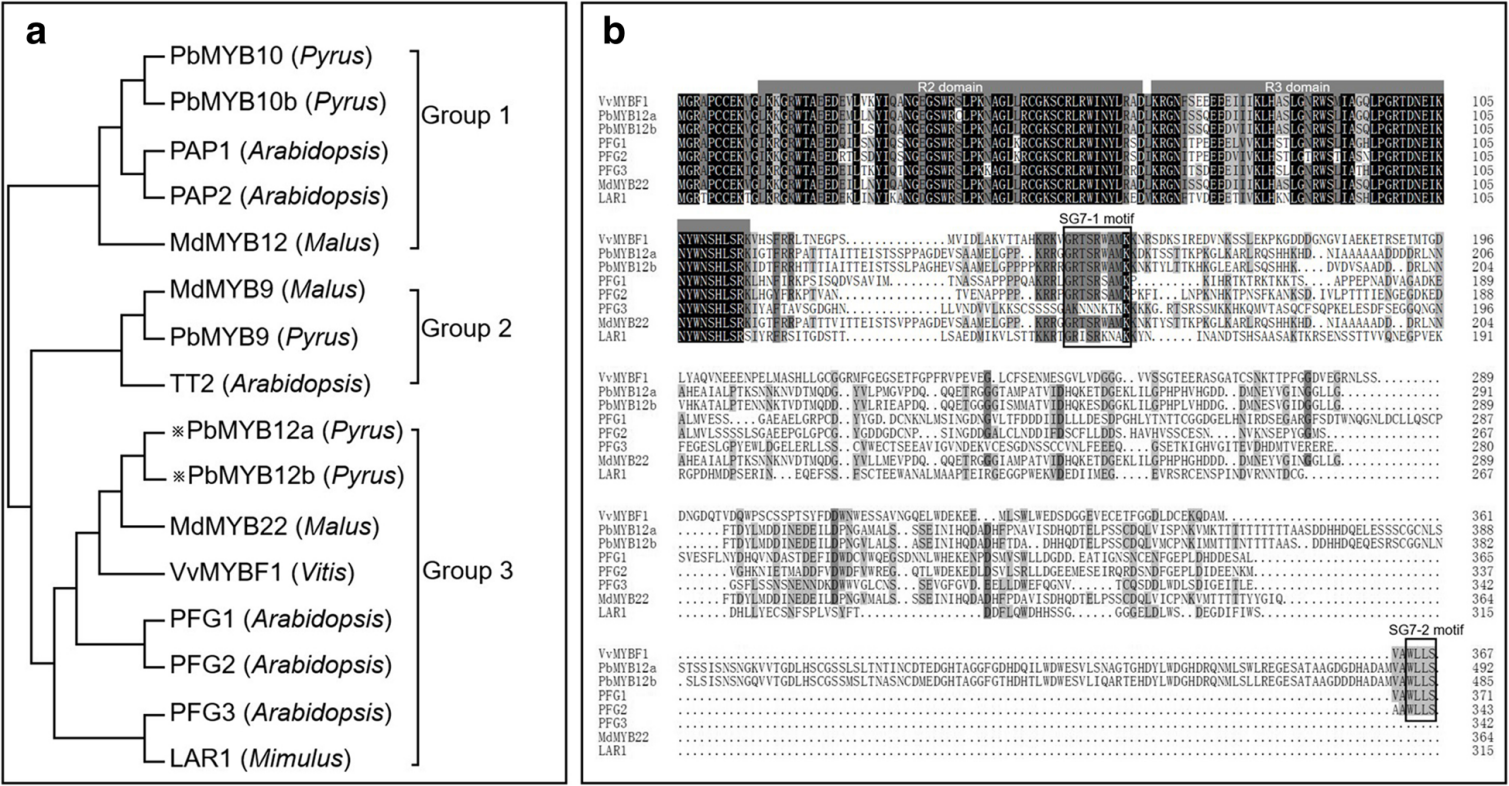
Sequence alignment and phylogenetic analysis of PbMYB12. **a** Phylogenetic analysis of typical flavonoid-regulating MYBs from different species. These MYBs were classified into three major groups. Pear PbMYB12a and PbMYB12b are marked by asterisks. The MYB protein sequences in other species were obtained from the NCBI database. Their protein accessions in the NCBI database are as follows: *Arabidopsis* PRODUCTION OF ANTHOCYANIN PIGMENT 1 (AEE33419.1), *Arabidopsis* PRODUCTION OF ANTHOCYANIN PIGMENT 2 (AAG42002.1), pear PbMYB10 (ALU57825.1), pear PbMYB10b (ALU57826.1), apple MdMYB12 (ADL36755.1), apple MdMYB9 (ABB84757.1), pear PbMYB9 (ALU57827.1), *Arabidopsis* TT2 (OAO91653.1), grapevine VvMYBF1 (ACV81697.1), apple MdMYB22 (AAZ20438.1), *Mimulus* LAR1 (ALP48586.1), *Arabidopsis* PRODUCTION OF FLAVONOL GLYCOSIDES 1 (PFG1) (OAP07166.1), *Arabidopsis* PFG2 (OAP04683.1) and *Arabidopsis* PFG3 (OAO93308.1). The phylogenetic tree was constructed by MEGA7.0 using a bootstrap test of phylogeny with the Neighbor-joining test and default parameters. **b** Protein sequence alignments of PFG-type MYBs from different species. The conserved R2 and R3 domains are underlined. Previously described SG7–1 and SG7–2 motifs are boxed. Identical and similar amino acids are shaded. The multiple alignment was performed using ClustalW

The full-length coding sequences (CDS) of *PbMYB12a* and *PbMYB12b* were cloned from ‘Red Zaosu’ and ‘Zaosu’. The lengths of the proteins encoded by *PbMYB12a* and *PbMYB12b* were 492 and 485 amino acids, respectively. Moreover, no variations in protein sequences were identified among ‘Red Zaosu’, ‘Zaosu’ and the pear reference genome. Typical MYB TFs involved in flavonoid biosynthesis could be classified into three different groups. PAP-type MYB TFs, including *PbMYB10* and *PbMYB10b* in pear, were identified as anthocyanin regulators and placed in group I. TT2-type MYB TFs, including *PbMYB9* in pear, were identified as flavanol regulators and placed in group II. Notably, *PbMYB12a* and *PbMYB12b* were clustered with PFG-type MYB TFs and placed into group III (Fig. 3a). A protein sequence alignment analysis showed that PbMYB12a and PbMYB12b contain two characteristic motifs, SG7–1 and SG7–2, which belong to PFG-type MYB TFs (Fig. 3b). Thus, PbMYB12a and PbMYB12b were identified as typical PFG-type MYB TFs having the potential capacity to regulate flavonol biosynthesis in pear fruit.

### Sub-cellular localization of PbMYB12b

**Fig. 4:**
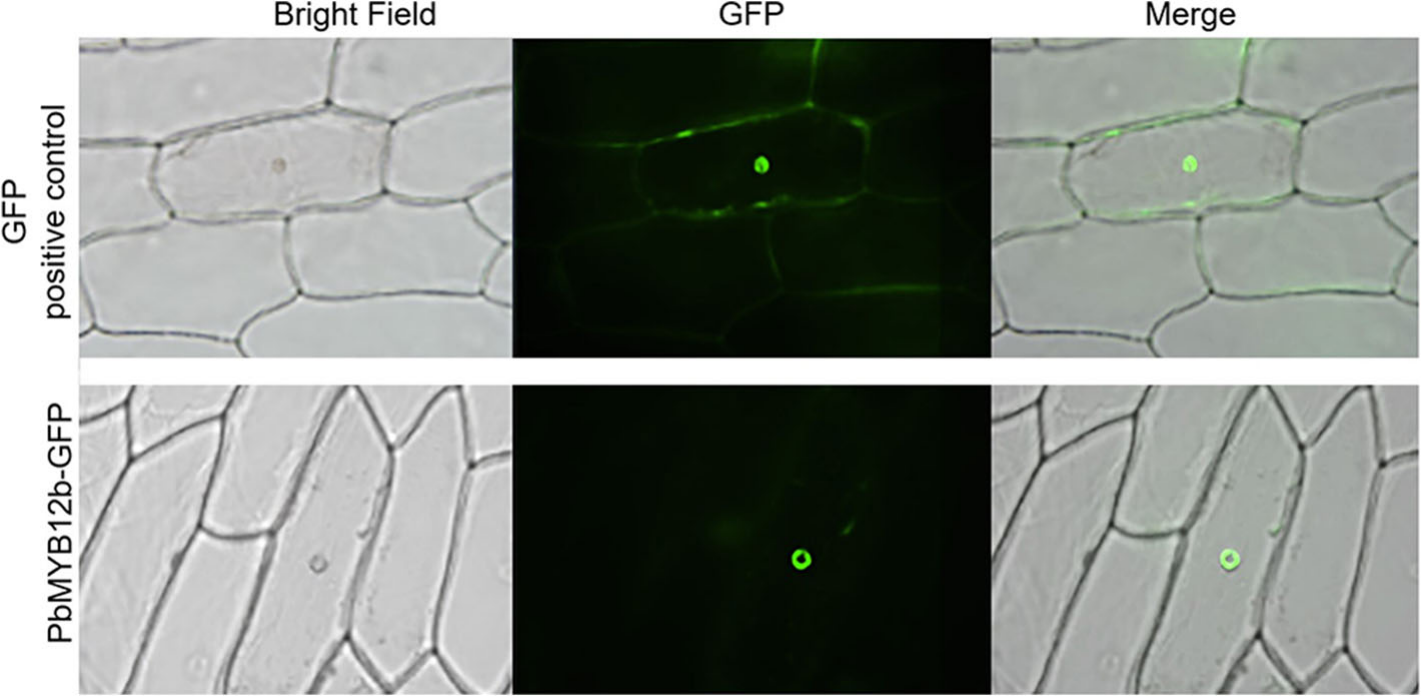
Sub-cellular localization of PbMYB12b in onion epidermal cells. The fusion protein (PbMYB12b-GFP) and GFP-positive control were independently transiently expressed in onion epidermal cells. GFP fluorescence was observed with a fluorescence microscope

The sub-cellular localization of PbMYB12b was inferred from the transient expression of *p35S::green fluorescent protein (GFP)-PbMYB12b* in onion epidermal cells. The transformed cells expressed *GFP* strictly in the nuclei (Fig. 4), whereas in control transgenic cells expressing p*35S::GFP*, the GFP signal was detected throughout the cells (Fig. 4).

### The *PbMYB12b* expression pattern was correlated with flavonol glycosides biosynthesis

**Fig. 5:**
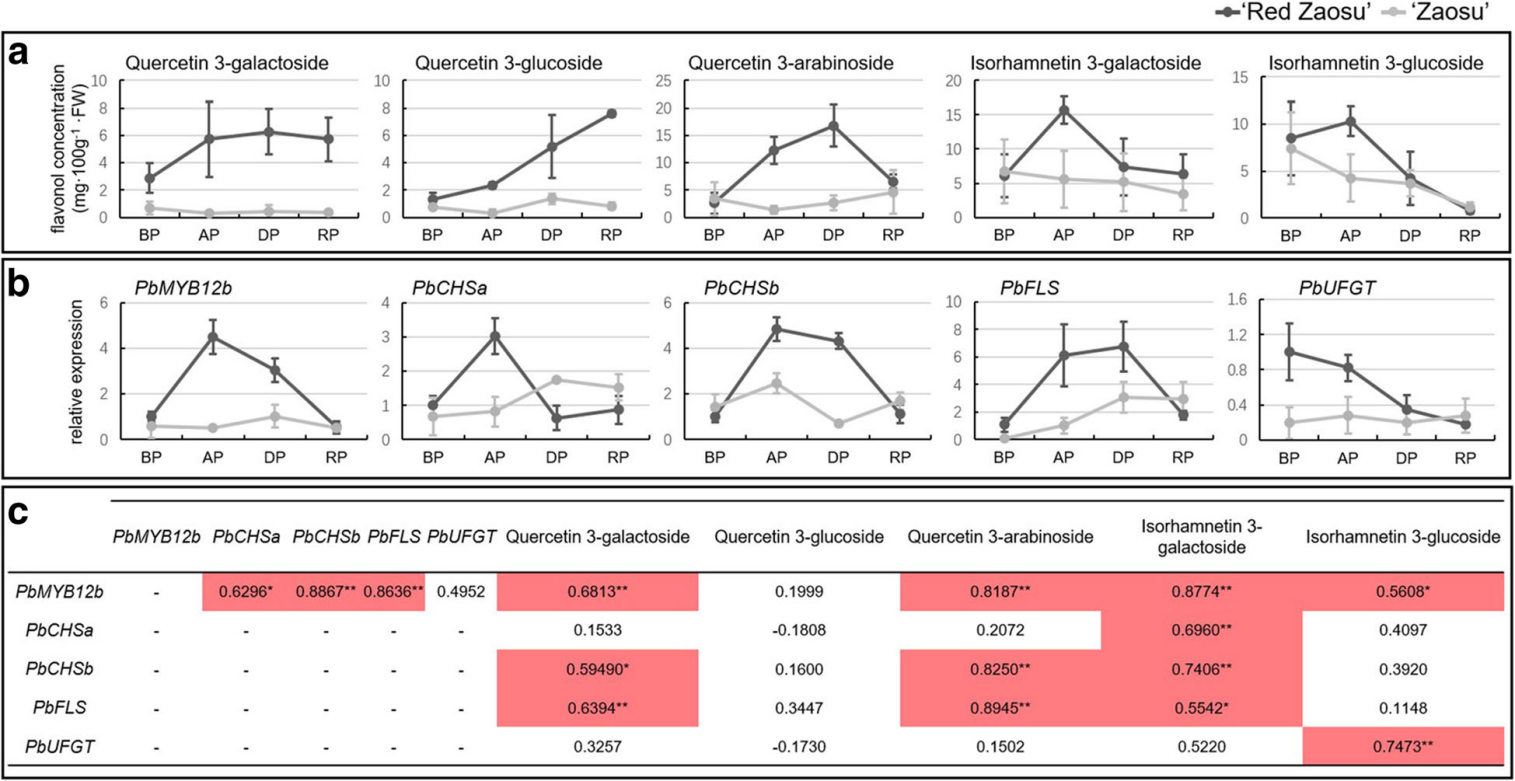
The correlation analysis between *PbMYB12b* and flavonol glycoside biosynthesis. **a** The concentrations of different flavonol glycosides in ‘Red Zaosu’ and ‘Zaosu’ fruit during four representative growth periods, before pollination (BP), after pollination (AP), developing period (DP) and ripening period (RP). **b** Expression analysis of *PbMYB12b* and flavonol biosynthetic genes during the four fruit growth periods using qRT-PCR. **c** The Pearson correlation coefficients of the expression patterns of *PbMYB12b* and flavonol biosynthetic genes, and the concentrations of different flavonol glycosides, during the four fruit growth periods. Asterisks indicate confidence levels ≥95% (*) and 99% (**)

The accumulation rates of quercetin 3-galactoside, quercetin 3-arabinoside and isorhamnetin 3-galactoside accelerated after pollination (AP) and slowed down when the fruit in ripening period (RP) in ‘Red Zaosu’ (Fig. 5a). The concentrations of quercetin 3-galactoside and quercetin 3-glucoside remained greater in RP fruit of ‘Red Zaosu’ than in ‘Zaosu’ (Fig. 5a). The expression pattern of *PbMYB12b* was positively correlated with most flavonol glycosides, except for quercetin 3-glucoside (Fig. 5b and c). Moreover, the expression pattern of *PbMYB12b* was positively correlated with the expression patterns of *PbCHSa*, *PbCHSb* and *PbFLS*, especially the latter two during fruit growth (Fig. 5b and c). Thus, *PbMYB12b* may positively regulate flavonol glycoside accumulation, and its putative target genes are *PbCHSa*, *PbCHSb* and *PbFLS.*

### *PbMYB12b* could induce flavonol accumulation in pear fruit

The validity of the infection on the leaves and fruit was proved by monitoring the GUS signals (Additional file 1: Figure S3a). Besides, in comparison with the non-infiltrated fruit and leaves, the flavonols concentration or the flavonoid biosynthesis genes did not change too much in those infiltrated fruit and leaves (Additional file 1: Figure S3a).

**Fig. 6:**
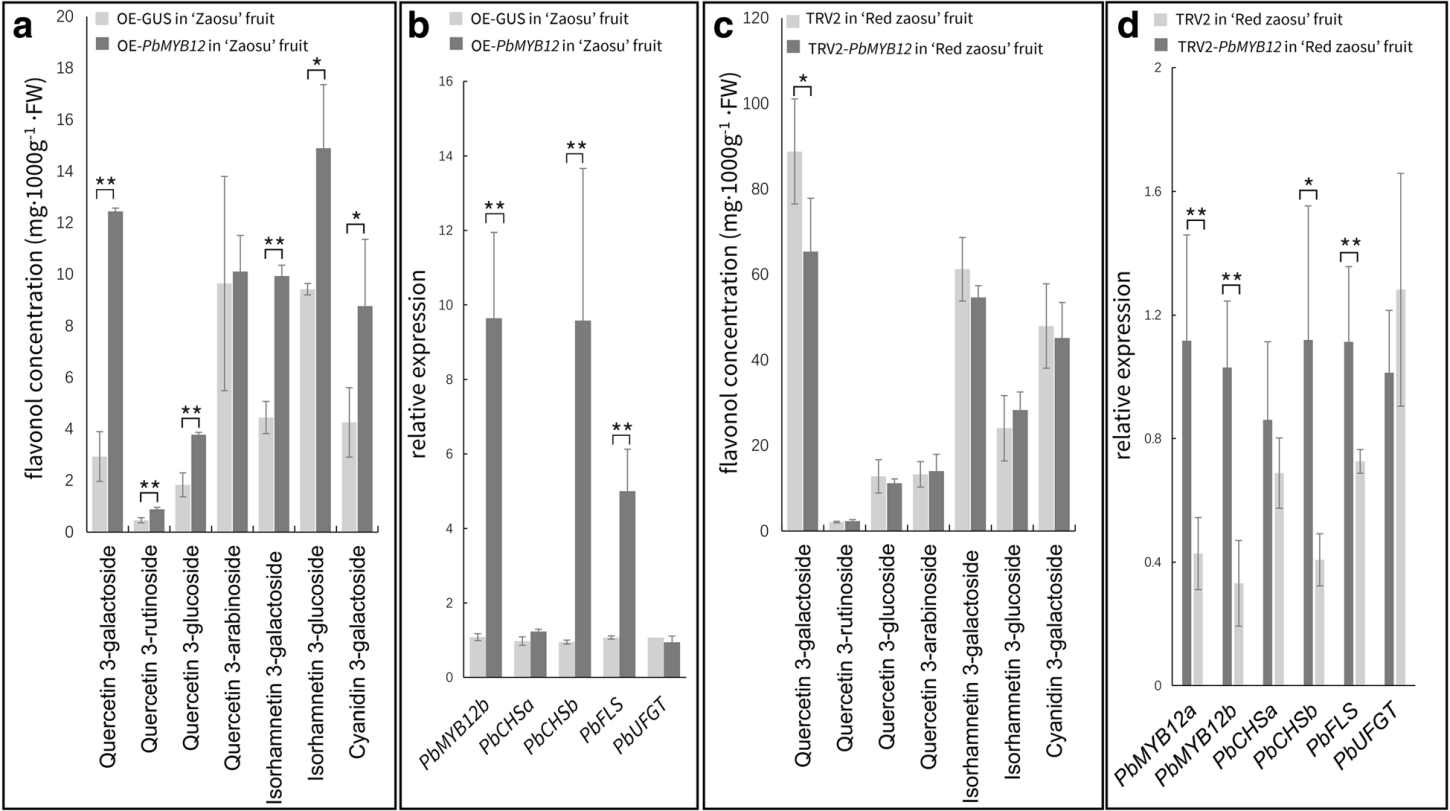
*PbMYB12b*-regulated flavonol biosynthesis in pear fruit. **a** Flavonol glycoside contents in ‘Zaosu’ fruit transiently overexpressing *PbMYB12b*. **b** The expression patterns of *PbMYB12b* and flavonol biosynthetic genes in ‘Zaosu’ fruit transiently overexpressing *PbMYB12b*. **c** Flavonol glycoside contents of ‘Red Zaosu ‘fruit transiently silencing *PbMYB12b* using VIGS. **d** The expression patterns of *PbMYB12b* and flavonol biosynthetic genes in ‘Red Zaosu’ fruit transiently silencing *PbMYB12b* using VIGS. The pear fruit in DP were infiltrated and sampled at 48 h after infiltration. The control fruit were infiltrated by pCambia1301-GUS (OE-GUS) and original TRV2, respectively. Data are the means ± SDs of three biological replicates. Asterisks indicate significant differences as assessed by Student’s t test: ^*^P < 0.05, ^**^P < 0.01

Transient overexpression of *PbMYB12b* in wild-type ‘Zaosu’ fruit revealed its specific function in regulating flavonol accumulation. The concentrations of most flavonol glycosides were up-regulated by the overexpression of *PbMYB12b*, except for quercetin 3-arabinoside (Fig. 6a). Unexpectedly, the accumulation of the major anthocyanin compound in pear fruit, cycanidin 3-galactoside, was also induced by the overexpression of *PbMYB12b* (Fig. 6a). For those flavonol biosynthetic genes, the overexpression of *PbMYB12b* up-regulated the expression of *PbCHSb* and *PbFLS*. Moreover, the expression patterns of *PbCHSa* and *PbUFGT* were not affected by *PbMYB12b* (Fig. 6b). The expression levels of *PbCHSb* and *PbFLS* were reduced when *PbMYB12b* expression was transcriptionally interrupted using a virus-induced gene silencing (VIGS) assay in ‘Red Zaosu’ fruit (Fig. 6d). The concentration of quercetin 3-galactoside, a major flavonol compound in pear fruit, was reduced in ‘Red Zaosu’ fruit having a low *PbMYB12b* expression level (Fig. 6c), but the concentrations of other flavonols were not reduced (Fig. 6d). This phenomenon could be explained by the long period of flavonol glycoside accumulation during DP, while the RNAi effect of VIGS was probably transient.

**Fig. 7:**
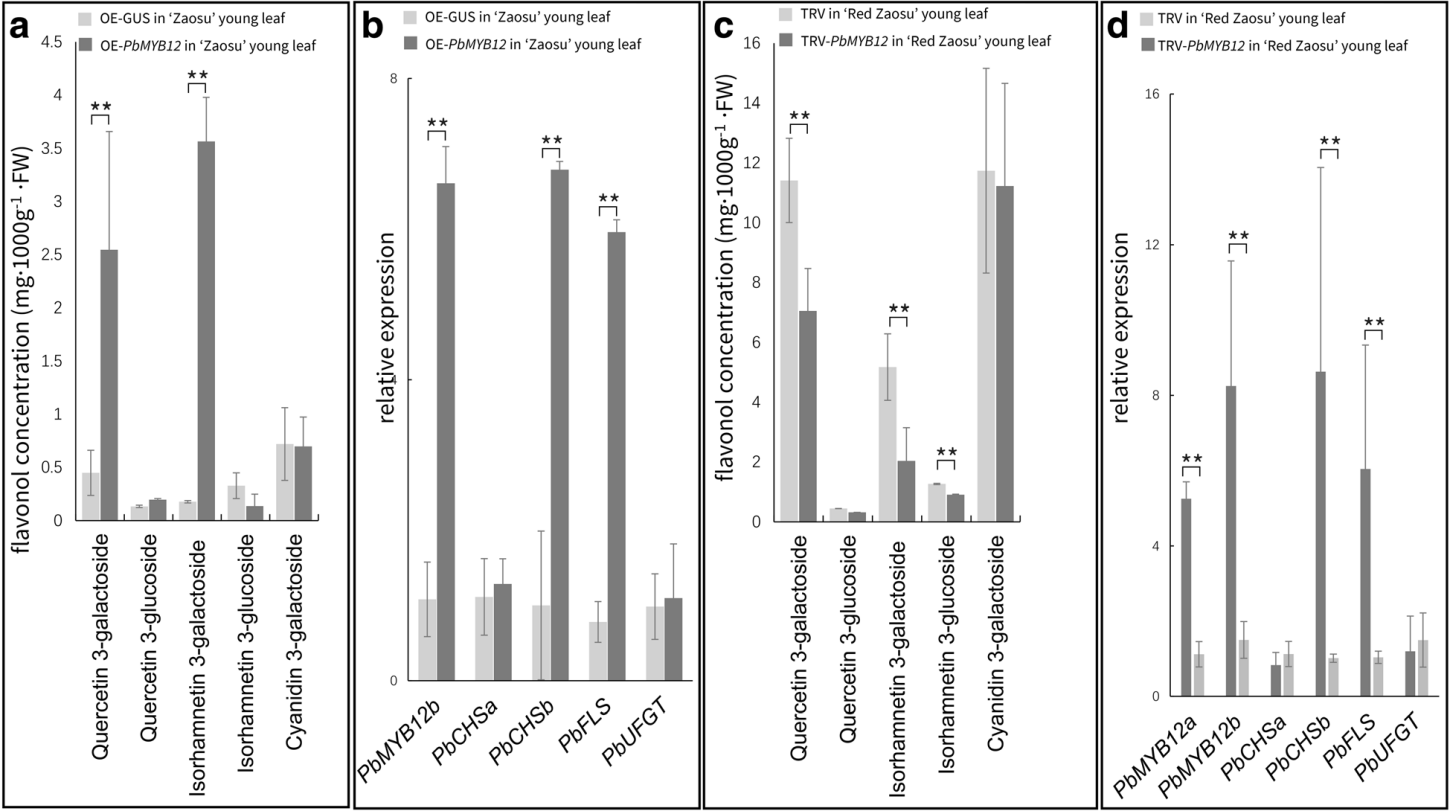
*PbMYB12b*-regulated flavonol biosynthesis in young pear leaves. **a** Flavonol glycoside contents in young ‘Zaosu’ leaves transiently overexpressing *PbMYB12b*. **b** The expression patterns of *PbMYB12b* and flavonol biosynthetic genes in young ‘Zaosu’ leaves transiently overexpressing *PbMYB12b*. **c** Flavonol glycoside contents in young ‘Red Zaosu’ leaves transiently silencing *PbMYB12b* using VIGS. **d** The expression patterns of *PbMYB12b* and flavonol biosynthetic genes in young ‘Red Zaosu’ leaves transiently silencing *PbMYB12b* using VIGS. The three-day-old leaves of tissue cultured pear plants were infiltrated and sampled at 48 h after infiltration. The control leaves were infiltrated by pCambia1301-GUS (OE-GUS) and original TRV2, respectively The DP fruit infiltrated by pCambia1301-GUS (OE-GUS) was the control. Data are the means ± SDs of three biological replicates. Asterisks indicate significant differences as assessed by Student’s t test: ^*^P < 0.05, ^**^P < 0.01

Therefore, to identify whether *PbMYB12b* regulates the biosynthesis of most flavonol compounds in pear, we further transient overexpressed and interrupted *PbMYB12b* expression in the unexpanded young leaves of ‘Zaosu’ and ‘Red Zaosu’ by vacuum infiltration, respectively. Quercetin 3-rubinoside and arabinoside were not identified in the young leaves of ‘Zaosu’ and ‘Red Zaosu’. The young ‘Zaosu’ leaves overexpressing *PbMYB12b* accumulated more quercetin 3-galactoside and isorhamnetin 3-galacotside than control leaves (Fig. 7a). The expression levels of *PbCHSb* and *PbFLS* were also up-regulated by the overexpression of *PbMYB12b* (Fig. 7b)*.* The biosynthesis of most flavonol glycosides in young leaves of ‘Red Zaosu’ was inhibited when *PbMYB12b* expression was interrupted (Fig. 7c). *PbCHSb* and *PbFLS* were also lowly expressed in these leaves (Fig. 7d). This result indicated that the regular expression of PbMYB12b was necessary in flavonol glycoside biosynthesis for pears.

Thus, the biofunction of *PbMYB12b* in flavonol biosynthetic regulation was verified, and two downstream genes of *PbMYB12b*, *PbCHSb* and *PbFLS*, were investigated*.*

### *PbMYB12b* activates the expression of *PbCHSb* and *PbFLS*

**Fig. 8:**
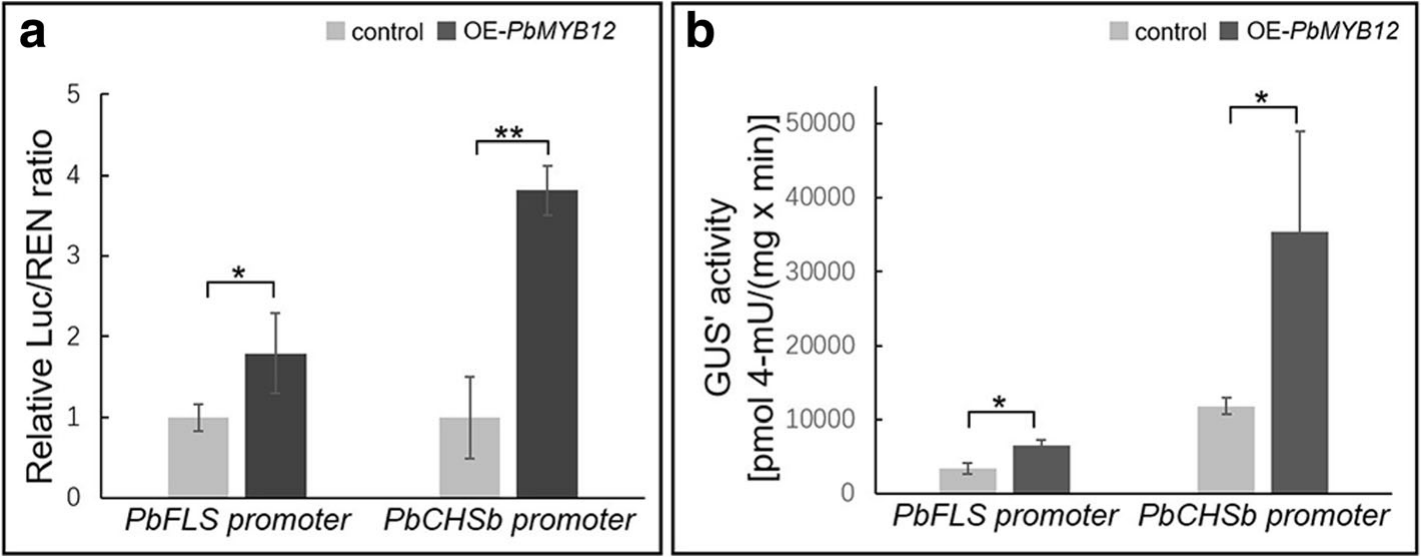
*PbMYB12b* activates *PbFLS* and *PbCHSb* promoters. **a** *PbMYB12b* activates *PbFLS* and *PbCHSb* promoters as assessed by the transient dual-luciferase expression assay. **b** *PbMYB12b* activates *PbFLS* and *PbCHSb* promoters as assessed by the transient GUS reporter assay. Data are the means ± SDs of five biological replicates. Asterisks indicate significant differences as assessed by Student’s t test: ^*^P < 0.05, ^**^P < 0.01

To determine whether *PbMYB12b* activates the expression of *PbCHSb* and *PbFLS*, we performed dual-luciferase (LUC) assays in tobacco leaves. The promoter regions of *PbCHSb* and *PbFLS* were amplified, independently transformed into the pGreenII 0800-LUC vector and co-infiltrated into tobacco leaves with overexpressing *PbMYB12b*. When *PbMYB12* was independently co-transformed with *PbCHSb* and *PbFLS* pro-LUC reporters, the LUC/ Renilla LUC (REN) ratios significantly increased compared with the empty control (Fig. 8a). This was consistent with our observations of GUS activity levels when *PbMYB12b* was co-expressed with the GUS reporter containing the independent promoter regions of *PbCHSb* and *PbFLS* (Fig. 8b). Thus, *PbMYB12b* positively regulate the expression patterns of *PbFLS* and *PbCHSb* by activating their promoters.

## Discussion

Flavonol glycosides are a group of flavonoid metabolites in plants that have potential health benefits for humans. There are various kinds of flavonol glycosides in different plant species. Quercetin glycosides are common flavonol compounds in fleshy fruit, including apple, pear and grape berry [3, 9, 19, 20]. Nevertheless, as a common fruit, pear has a unique compositions of flavonol glycosides owing to the accumulation isorhamnetin glycosides [9, 21, 22]. Although the biosynthetic enzymes of flavonoid compounds have been investigated in pears, neither the biosynthetic pathway of flavonols, nor their regulatory networks, have been specifically studied [3]. Previously, we reported that UFGT was a key biosynthetic enzyme in the accumulation of flavonoid glycosides, including flavonols [9]. In the present study, the expression patterns of the genes encoding CHS and FLS were consistent with the accumulation patterns of quercetin glycosides and isorhamnetin glycosides (Fig. 5). The roles of CHS and FLS in flavonol biosynthesis have been reported in other plants. The expression of the *CHS* gene is deficient in the *Arabidopsis* mutant *tt4*, and flavonols cannot accumulate in the mutant *tt4* [23]. FLS exclusively catalyzes flavonol glycoside biosynthesis, and the regular expression of the *FLS* gene is necessary for flavonol accumulation in *Arabidopsis* seedlings [24]. Thus, the biosynthesis of flavonol glycosides may not be determined by a single biosynthetic enzyme but may be influenced by a series of biosynthetic enzymes, including CHS, FLS and UFGT.

In this study, a MYB TF was identified as being involved in regulating flavonol glycoside biosynthesis in pear fruit. *PbMYB12b* is a typical PFG-type MYB TF, and its expression pattern is consistent with the flavonol accumulation pattern in pear fruit (Figs. 3 and 5). *PbMYB12b* largely expressed in young ‘Red Zaosu’ fruit (AP) and kept decreasing during the fruit development (DP and RP), this result is also consistent with the expression patterns of *PbMYB12* in ‘Dangshansu’ pear fruit during its development [25]. Overexpressed *PbMYB12b* induced the biosynthesis of most quercetin glycosides and isorhamnetin glycosides in pear fruit (Fig. 6). In apple calli, the overexpression of *MdMYB22*, a PFG-type MYB TF, also induces flavonol accumulation but reduces anthocyanin biosynthesis [20]. However, in our study, the anthocyanin concentration increased in ‘Zaosu’ fruit overexpressing *PbMYB12b* but was not decreased in ‘Red Zaosu’ fruit with interrupted *PbMYB12b* (Fig. 6). The result indicated that *PbMYB12b* could be a positive regulator of anthocyanin biosynthesis but not the dominant or necessary TF of anthocyanin biosynthesis.

Transient silenced *PbMYB12b* by VIGS only reduced the concentration of quercetin 3-galactoside in pear fruit skin (Fig. 6c and d). However more flavonol glycosides were significantly affected by *PbMYB12b* silencing in leaves (Fig. 7c and d). This result indicated that *PbMYB12b* is more functional in leaves and probably not fruit specific. Besides, levels of flavonol glycosides in leaves are in fact lower in comparison with fruits (Fig. 2, Additional file 1: Figure S2). The leaves with less flavonols accumulation may provide a more sensitive environment to detect the effects of transient silenced *PbMYB12b*. Although the flavonol biosynthetic related genes were suppressed by silenced *PbMYB12b*, the high level flavonol background in DP fruit maybe still overshadowed the effect of silenced *PbMYB12b* on the flavonol concentration level. The low flavonols background in leaves also could explain why more flavonol glycosides were significantly affected by *PbMYB12b* silencing in leaves.

*MdMYB22* specifically up-regulates the expression of *MdFLS* but down-regulates that of *MdCHS* in apple. *VvMYBF1*, a PFG-type MYB TF in grapevine, activates the expression of CHS and FLS by binding their promoters [19]. In the present study, we verified that PbMYB12b positively regulated the expression of *PbCHSb* and *PbFLS* by activating their promoters (Fig. 8a and b). Interestingly, *PbMYB12b* and *VvMYBF1* both have a conserved SG7–2 motif with four residues, WLLS, that is not found in *MdMYB22* (Fig. 3). This observation indicated that there were two types of PFG MYB TFs that both induced flavonol accumulation but one could inhibit anthocyanin biosynthesis at the same time. The correlations between the sequence variations in the two types of PFG MYB TFs and their biofunctions in different regulatory networks for flavonol biosynthesis will be studied in the future.

## Conclusions

Based on our results, *PbMYB12b*’s regulation of flavonol biosynthesis was investigated. *PbMYB12b* could up-regulate the expression levels of *PbCHSb* and *PbFLS*, and their high-level expression resulted in the accumulation of flavonol compounds, including quercetin glycosides and isorhamnetin glycosides, in pear fruit. *PbMYB12b* also contributes slightly to the accumulation of anthocyanin in pear fruit. An increased understanding of regulatory networks for flavonol biosynthesis in pear fruit could help to breed flavonoid-enriched cultivars, providing nutritious fruit.

## Materials and methods

### Plant materials and growing conditions

In the present study, ‘Red Zaosu’ (*P. bretschneideri* Rehd.) and ‘Zaosu’ (*P. bretschneideri* Rehd.) were collected from the Horticultural Research Base of Northwest A&F University in Yangling Distinct, Shaanxi Province, China. The pear fruit were harvested and sampled at four representative points during the whole-fruit growth period, including 5 d before pollination (BP), 15 d after pollination (AP), 50 d after pollination (DP) and 110 d after pollination (fruit ripen period, RP). Moreover, the pear fruit in DP and three-day-old leaves of tissue cultured pear plants were selected to transiently verify the bio-funtion of *PbMYB12b*. The detailed experimental design was illustrated in Additional file 1: Figure S1.

Fresh plant tissues for later use were immediately frozen, ground to a powder in liquid nitrogen and stored at − 80 °C.

### DNA and RNA extraction and purification

Total DNA and RNA were extracted and purified by SDS solubilization and phenol extraction [26].

### RNA-seq and analysis

During the DP, 3 μg total RNA were extracted from receptacles and fruit skins of ‘Red Zaosu’ and ‘Zaosu’, with three biological replicates. These were used for sequencing. Briefly, total RNA was randomly fragmented, reversed-transcribed, amplified and purified to form cDNA libraries. After the assessment of the cDNA libraries using an Agilent Bioanalyzer 2100 system, the library preparations were paired-end sequenced (100 bp) on an Illumina HiSeq2500 platform. Clean reads were screened by removing reads containing adapters and poly-Ns, and low-quality reads, from the raw data. Paired-end clean reads were aligned to the pear genome using TopHat [27]. Genes with q-values < 0.05, as assessed by the DESeq R package, and fold changes > 2 were assigned as differentially expressed genes between ‘Red Zaosu’ and ‘Zaosu’ [28].

### RT-qPCR

In total, 2 μg purified RNA was reverse-transcribed to cDNA using the PrimeScript RT Reagent Kit with gDNA Eraser (TaKaRa). The primer pairs of selected genes and *PbActin* (internal control) are listed in Additional file 1: Table S2. PCR reactions were performed on a StepOnePlus PCR system (Applied Biosystems) with SYBR Premix Ex Taq II (TaKaRa) according to the manufacturer’s instructions. Expression data of three biological replicates were analyzed according to the cycle threshold 2^−ΔΔCt^ method.

### Transient assay

The transient expression assays were performed as previously described, with a modified infiltration method [15]. For the overexpression of *PbMYB12b*, the full-length *PbMYB12b* CDS was PCR-amplified from ‘Red Zaosu’ cDNA sources and then replaced the GFP-coding sequence in the pCambia 1301 binary vector using ClonExpress One Step Cloning Kit (Vazyme) based on homologous recombination technology. For the RNAi of *PbMYB12b* using VIGS, a 479-bp fragment of the *PbMYB12b* CDS was PCR-amplified from ‘Red Zaosu’ cDNA sources and then inserted into the MCS region in the pTRV2 vector using a ClonExpress One Step Cloning Kit (Vazyme) based on homologous recombination technology. For the record, the non-specific silencing of *PbMYB12a* is inevitable. The mRNA sequence homology between *PbMYB12a* and *PbMYB12b* were 89%, and for the 479-bp fragment were 92%. The plasmid was transferred into the *Agrobacterium tumefaciens* strain GV3101. The Agrobacterium cells containing pCambia 1301-GUS, pCambia 1301-PbMYB12b, pTRV2-PbMYB12b, original pTRV2 and pTRV1 were suspended at 28 °C in LB medium containing 10 mM MES and 20 μM acetosyringone with appropriate antibiotics, respectively. *Agrobacterium* cells were harvested and resuspended for 4 h in infiltration buffer (10 mM MgCl_2_, 10 mM MES (pH 5.6) and 100 μM acetosyringone) to a final OD_600_ = 0.8 at room temperature before infiltration. Fruit infiltration was performed by injecting 1 mL *Agrobacterium* cells. Leaf infiltration was performed by vacuuming instead of injection. Young leaves (three-day-old) of ‘Zaosu’ and ‘Red Zaosu’ explants that cultured in 1/2 MS solid medium (pH = 5.5) were totally immersed and vacuumed in 2 mL *Agrobacterium* suspensions at 25 kPa for 5 min. For transient overexpression, the DP fruit and young leaves of ‘Zaosu’ infiltrated by pCambia 1301-PbMYB12b were sampled at 48 h after infiltration, the DP fruit and young leaves of ‘Zaosu’ infiltrated by pCambia 1301-GUS and the un-infiltrated DP fruit and leaves were also sampled as controls. For transient RNAi, the DP fruit and young leaves of ‘Red Zaosu’ co-infiltrated by pTRV2-PbMYB12b and pTRV1 were sampled at 48 h after infiltration, the DP fruit and young leaves of ‘Red Zaosu’ co-infiltrated by pTRV2 and pTRV1 and the un-infiltrated DP fruit and leaves were also sampled as controls. For GUS staining, the plant materials were stained with 5-bromo-4-chloro-3-indolyl glucuronide (X-Gluc) at 37 °C for 12 as described [29]. The primer pairs are listed in Additional file 1: Table S2.

### Analysis of flavonoid compounds

The extraction and analysis of flavonoid compounds were carried out as previously described [30]. Briefly, the flavonoids were extracted with 70% methanol containing 2% formic acid at 0–4 °C. The supernatant was filtered through a 0.45-μm syringe filter prior to HPLC analysis. Phenolic compounds were analyzed using a HP1200 Liquid Chromatograph equipped with a diode array detector (Agilent Technology, Palo Alto, CA, USA). An Inertsil ODS-3 column (5.0 μm, 4.0 × 250 mm, GL Sciences Inc., Tokyo, Japan) was used in the separation, preceded by an Inertsil ODS-3 Guard Column (5.0 μm, 4.0 × 10 mm). Solvent A consisted of 10% formic acid (11.36% of 88% formic acid) dissolved in water, and solvent B was 10% formic acid (11.36% of 88% formic acid) and 1.36% water in acetonitrile (HPLC grade, purity: 99.9%). The gradient was 95% solvent A (0 min), 85% solvent A (25 min), 78% solvent A (42 min), 64% solvent A (60 min) and 95% solvent A (65 min). The post-run-time was 10 min. The flow rate was 1.0 mL min^− 1^ at 30 °C. Simultaneous monitoring was performed at 365 nm for quercetin-3-galactoside, quercetin-3-glucoside, quercetin-3-arabinoside, quercetin-3-rutinoside, isrohamnetin-3-galactoside and isrohamnetin-3-gluctoside, and at 520 nm for cyanidin-3-galactoside. Peaks were identified by a comparison of retention times and UV spectra with those of authentic standards. The concentrations of individual phenolic compounds were determined based on peak areas and calibration curves that were derived from the corresponding authentic phenolic compounds. All of the phenolic standards were obtained from Sigma Aldrich (St Louis, MO, USA), Extrasynthese (Genay Cedex, France) and AApin Chemicals (Abingdon, Oxon, UK).

### Sub-cellular localization of the PbMYB12b protein in the onion epidermis

The full-length *PbMYB12b* CDS was amplified and fused into pCambia 2300 to generate the transgene construct p*35S::GFP-PbMYB12b* using a ClonExpress One Step Cloning Kit (Vazyme) based on homologous recombination technology. The construct was transiently introduced into onion epidermal cells by injection, and control cells were transformed in the same way with p*35S::GFP*. After their transformation, the onion epidermal samples were held for 16 h at 25 °C in the dark, after which GFP fluorescence was imaged using a BX51 + PD72 + IX71 microscopic imaging system (OLYMPUS).

### Dual-LUC reporter assays in tobacco leaves

To assay the effects of *PbMYB12b* on *PbCHSb* and *PbFLS*, their promoters were amplified and inserted into pGreenII 0800-LUC double-reporter plasmid as reporters (primer pairs are listed in Additional file 1: Table S2) [31]. The effector plasmid was constructed by inserting *PbMYB12b* into the p62-SK vector. The constructed effector and reporter plasmids, in different combinations, were co-transformed into tobacco leaves.

The activities of LUC and REN were quantified 48 h after infiltration with a dual LUC assay kit (Promega) using a Luminoskan Ascent Microplate Luminometer (Thermo Fisher Scientific). The transcriptional capability of *PbMYB12* was assessed by the LUC/REN ratio. Five biological repeats were included for each pair.

### GUS reporter assays in tobacco leaves

To assay the effects of *PbMYB12b* on *PbCHSb* and *PbFLS*, their promoters were individually inserted into the pBI121-GUS plasmid as reporters. An effector plasmid was constructed by inserting *PbMYB12b* into the p62-SK vector. The constructed effector and reporter plasmids, in different combinations, were co-transformed into tobacco leaves. The GUS activity was measured as described previously [29].

### Statistical analysis

To determine significant differences among the data, Student’s t test was conducted using SPSS 16.0.

## Additional file

Additional file 1: **Figure S1.** The illustration of experimental design. **Figure S2.** Flavonol glycoside concentrations and the expression patterns of related genes in tissue cultured pear leaves. **Figure S3.** The transient efficiency in pear fruit and leaves. **Table S1.** FPKM values of flavonol biosynthesis related genes. (DOCX 1624 kb)

## Abbreviations

AP: After pollination
AP: After pollination
BP: Before pollination
CHI: Chalcone isomerase
CHS: Chalcone synthase
DP: Developing period
F3H: Flavanone 3-hydroxylase
FLS: Flavonol synthase
PAP: PRODUCTION OF ANTHOCYANIN PIGMENT
PFG: PRODUCTION OF FLAVONOL GLYCOSIDES
QOMT: Quercetin 3-O-methyltransferase
RP: Ripen period
TF: Transcription factor
TT2: TRANSPARENT TESTA 2
UFGT: UDP-glucose: flavonoid 3-glucosyltransferase
VIGS: Virus induced gene silencing

## References

[1] Halbwirth H (2010). The creation and physiological relevance of divergent hydroxylation patterns in the flavonoid pathway. Int J Mol Sci.

[2] Harnly JM, Doherty RF, Beecher GR, Holden JM, Haytowitz DB, Bhagwat S, Gebhardt S (2006). Flavonoid content of U.S. fruits, vegetables, and nuts. J Agric Food Chem.

[3] Fischer TC, Gosch C, Pfeiffer J, Halbwirth H, Halle C, Stich K, Forkmann G (2007). Flavonoid genes of pear (Pyrus communis). Trees..

[4] Yilmaz Y, Toledo RT (2004). Major flavonoids in grape seeds and skins: antioxidant capacity of catechin, epicatechin, and gallic acid. J Agric Food Chem.

[5] Li P, Ma F, Cheng L. Primary and secondary metabolism in the sun-exposed peel and the shaded peel of apple fruit. Physiol Plant. 2013;148:9–24. 10.1111/j.1399-3054.2012.01692.x 22989296

[6] Kuhn BM, Geisler M, Bigler L, Ringli C (2011). Flavonols accumulate asymmetrically and affect auxin transport in Arabidopsis. Plant Physiol.

[7] Forbes AM, Meier GP, Haendiges S, Taylor LP (2014). Structure–activity relationship studies of Flavonol analogues on pollen germination. J Agric Food Chem.

[8] Ylstra B, Busscher J, Franken J, Hollman PC, Mol JN, Van Tunen AJ (1994). Flavonols and fertilization in Petunia hybrida: localization and mode of action during pollen tube growth. Plant J.

[9] Zhai R, Liu XT, Feng WT, Chen SS, Xu LF, Wang ZG, Zhang JL, Li PM, Ma FW (2014). Different biosynthesis patterns among flavonoid 3-glycosides with distinct effects on accumulation of other flavonoid metabolites in pears (Pyrus bretschneideri Rehd.). PLoS One.

[10] Borevitz JO, Xia Y, Blount J, Dixon RA, Lamb C (2000). Activation tagging identifies a conserved MYB regulator of phenylpropanoid biosynthesis. Plant Cell.

[11] Gonzalez A, Zhao M, Leavitt JM, Lloyd AM (2008). Regulation of the anthocyanin biosynthetic pathway by the TTG1/bHLH/Myb transcriptional complex in Arabidopsis seedlings. Plant J.

[12] Nesi N, Jond C, Debeaujon I, Caboche M, Lepiniec L (2001). The Arabidopsis TT2 gene encodes an R2R3 MYB domain protein that acts as a key determinant for proanthocyanidin accumulation in developing seed. Plant Cell.

[13] Stracke R, Jahns O, Keck M, Tohge T, Niehaus K, Fernie AR, Weisshaar B (2010). Analysis of PRODUCTION OF FLAVONOL GLYCOSIDES-dependent flavonol glycoside accumulation in Arabidopsis thaliana plants reveals MYB11-, MYB12- and MYB111-independent flavonol glycoside accumulation. New Phytol.

[14] Espley RV, Hellens RP, Putterill J, Stevenson DE, Kutty-Amma S, Allan AC (2006). Red colouration in apple fruit is due to the activity of a MYB transcription factor, MdMYB10. Plant J.

[15] Zhai R, Wang Z, Zhang S, Meng G, Song L, Wang Z, Li P, Ma F, Xu L (2016). Two MYB transcription factors regulate flavonoid biosynthesis in pear fruit (Pyrus bretschneideri Rehd.). J Exp Bot.

[16] Gesell A, Yoshida K, Tran LT, Constabel CP (2014). Characterization of an apple TT2-type R2R3 MYB transcription factor functionally similar to the poplar proanthocyanidin regulator PtMYB134. Planta.

[17] An XH, Tian Y, Chen KQ, Liu XJ, Liu DD, Xie XB, Cheng CG, Cong PH, Hao YJ (2015). MdMYB9 and MdMYB11 are involved in the regulation of the JA-induced biosynthesis of anthocyanin and proanthocyanidin in apples. Plant Cell Physiol.

[18] Mehrtens F, Kranz H, Bednarek P, Weisshaar B (2005). The Arabidopsis transcription factor MYB12 is a flavonol-specific regulator of phenylpropanoid biosynthesis. Plant Physiol.

[19] Czemmel S, Stracke R, Weisshaar B, Cordon N, Harris NN, Walker AR, Robinson SP, Bogs J (2009). The grapevine R2R3-MYB transcription factor VvMYBF1 regulates flavonol synthesis in developing grape berries. Plant Physiol.

[20] Wang N, Xu H, Jiang S, Zhang Z, Lu N, Qiu H, Qu C, Wang Y, Wu S, Chen X (2017). MYB12 and MYB22 play essential roles in proanthocyanidin and flavonol synthesis in red-fleshed apple (Malus sieversii f. Niedzwetzkyana). Plant J.

[21] Bilia AR, Gonzalez JM, Morelli I, Nieri E, Rubio ME (1994). Flavonol glycosides from Pyrus bourgaeana. Phytochemistry.

[22] Nakajima M, Sakai Y, Oogaki C (1983). A species-specific flavonoid from Pyrus ussuriensis max. And Pyrus aromatica Nakai et Kikuchi, and its geographical distribution in Japan. Breeding Sci.

[23] Filkowski J, Kovalchuk O, Kovalchuk I (2004). Genome stability of vtc1, tt4, and tt5 Arabidopsis thaliana mutants impaired in protection against oxidative stress. Plant J.

[24] Nguyen NH, Kim JH, Kwon J, Jeong CY, Lee W, Lee D, Hong SW, Lee H (2016). Characterization of Arabidopsis thaliana FLAVONOL SYNTHASE 1 (FLS1) -overexpression plants in response to abiotic stress. Plant Physiol Biochem.

[25] Cao Y, Han Y, Li D, Lin Y, Cai Y (2016). MYB transcription factors in Chinese pear (Pyrus bretschneideri Rehd.): genome-wide identification, classification, and expression profiling during fruit development. Front Plant Sci.

[26] Jinjin Z, Yuejin W, Xiping W, Keqiang Y, Jinxiao Y (2003). An improved method for rapidly extracting total RNA from Vitis. J Food Sci.

[27] Trapnell C, Pachter L, Salzberg SL (2009). TopHat: discovering splice junctions with RNA-Seq. Bioinformatics.

[28] Anders S, Huber W (2010). Differential expression analysis for sequence count data. Genome Biol.

[29] Fillatti JJ, Kiser J, Rose R, Comai L (1987). Efficient transfer of a glyphosate tolerance gene into tomato using a binary agrobacterium tumefaciens vector. Biotechnology.

[30] Zhang Y, Li P, Cheng L. Developmental changes of carbohydrates, organic acids, amino acids, and phenolic compounds in ‘Honeycrisp’ apple flesh. Food Chem 2010;123**:**1013–1018 (2010).

[31] Hellens RP, Allan AC, Friel EN, Bolitho K, Grafton K, Templeton MD, Karunairetnam S, Gleave AP, Laing WA (2005). Transient expression vectors for functional genomics, quantification of promoter activity and RNA silencing in plants. Plant Methods.

