# Supplementary material for: The MYB transcription factor PbMYB12b positively regulates flavonol biosynthesis in pear fruit
Source: BMC Plant Biol. 2019 Feb 21;19:85. doi: 10.1186/s12870-019-1687-0 (PMC6385385; doi:10.1186/s12870-019-1687-0)
Supplement: Supplementary file 1 — Figure S1. The illustration of experimental design. Figure S2. Flavonol glycoside concentrations and the expression patterns of related genes in tissue cultured pear leaves. Figure S3. The transient efficiency in pear fruit and leaves. Table S1. FPKM values of flavonol biosynthesis related genes. (DOCX 1624 kb) [file 12870_2019_1687_MOESM1_ESM.docx]

**Figure S1 The illustration of experimental design**


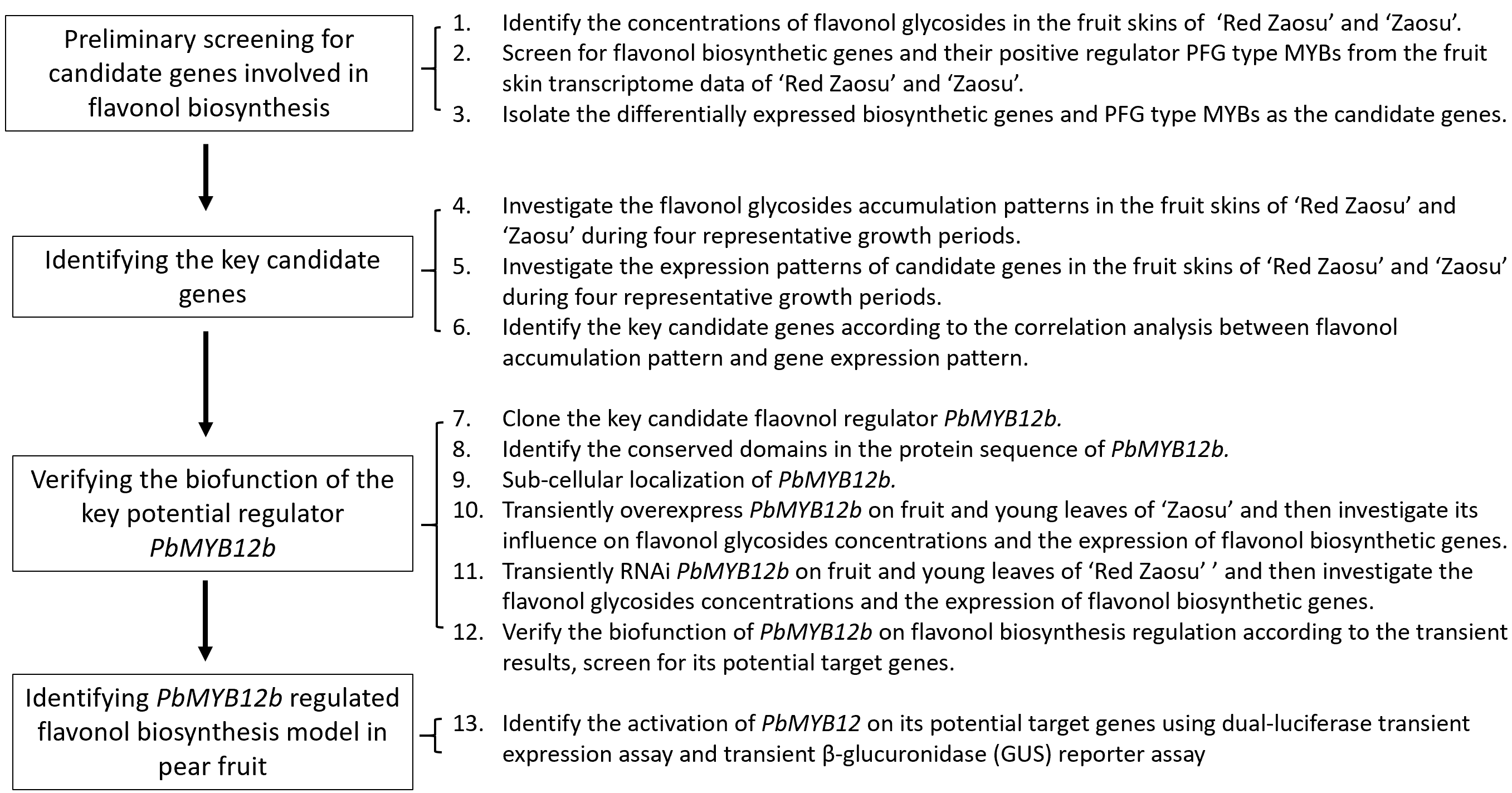


**Figure S2 Flavonol glycoside concentrations and the expression patterns of related genes in tissue cultured pear leaves**

a Flavonol glycoside concentrations in tissue cultured ‘Red Zaosu’ and ‘Zaosu’ leaves. Data are the means ± SDs of five biological replicates. Asterisks indicate significant differences between ‘Red Zaosu’ and ‘Zaosu’ as assessed by Student’s t test: **P < 0.01.

b The expression patterns of flavonol biosynthesis related genes in tissue cultured ‘Red Zaosu’ and ‘Zaosu’ leaves. Data are the means ± SDs of three biological replicates. Asterisks indicate significant differences between ‘Red Zaosu’ and ‘Zaosu’ as assessed by Student’s t test: **P < 0.01.

**
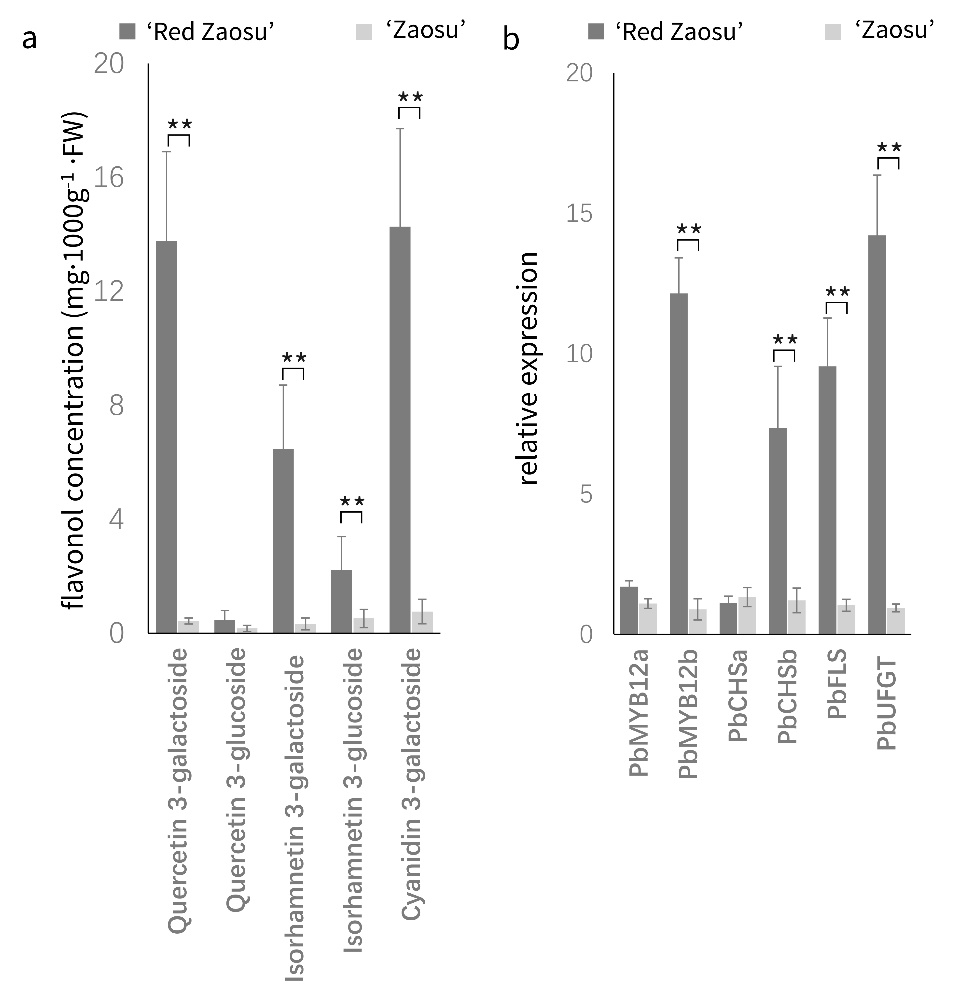
**

**Figure S3 The transient efficiency in pear fruit and leaves**

a. The GUS-stained ‘Zaosu’ leaves and DP fruit infiltrated by pCambia 1301-GUS

b. The ratios of flavonol biosynthetic genes expression and flavonol concentration between infiltrated and non-infiltrated fruit/leaves.

ND indicated non-detected. The significant differences were shaded by red, the non-significant differences were shaded by grey.


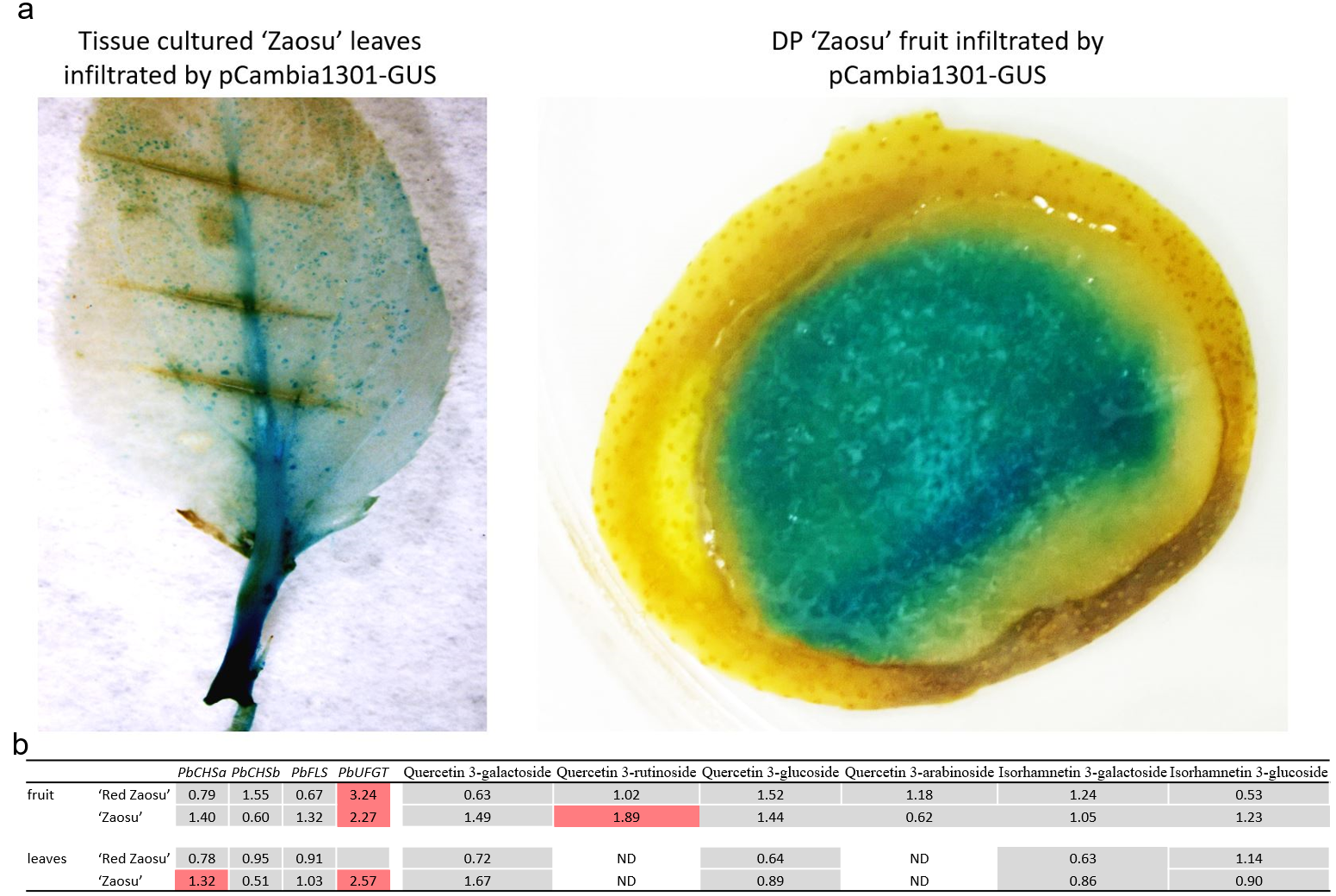


**Table S1** FPKM values of flavonol biosynthesis related genes

|  | Gene accessions | FPKM values of 'Red Zaosu' | | |  | FPKM values of 'Zaosu' | | |
| --- | --- | --- | --- | --- | --- | --- | --- | --- |
| PbMYB12a | LOC103967156 | 1.23463 | 0.65006 | 1.0525 |  | 0.334826 | 0.5159 | 0.520268 |
| PbMYB12b | LOC103931986 | 4.53434 | 4.67729 | 5.01637 |  | 0.473755 | 0.368036 | 0.257036 |
|  |  |  |  |  |  |  |  |  |
| Chalcone synthase | |  |  |  |  |  |  |  |
| - | LOC103945020 | 0.482913 | 0.270279 | 0.358998 |  | 0.234554 | 0.522759 | 0.270306 |
| - | LOC103945021 | 0.220697 | 0.0001 | 0.097887 |  | 0.0001 | 0.079319 | 0.0001 |
| - | LOC103959489 | 0.548432 | 0.0001 | 0.388329 |  | 0.0001 | 0.0001 | 0.0001 |
| PbCHSa | LOC103965055 | 7.71765 | 11.1953 | 11.2805 |  | 4.58666 | 6.69661 | 4.896 |
| PbCHSb | LOC103965056 | 10.0463 | 13.6507 | 13.4756 |  | 3.59632 | 3.87044 | 4.75954 |
| - | LOC103965057 | 0.361083 | 0.605575 | 0.561513 |  | 0.174754 | 0.902431 | 0.120983 |
|  |  |  |  |  |  |  |  |  |
| Chalcone isomerase | |  |  |  |  |  |  |  |
| - | LOC103928583 | 0.074478 | 0.18379 | 0.066063 |  | 0.0001 | 0.053026 | 0.04948 |
| PbCHI | LOC103936753 | 5.1982 | 5.65593 | 4.94159 |  | 5.06914 | 3.75613 | 4.37306 |
|  |  |  |  |  |  |  |  |  |
| Flavanone-3-hydroxylase | |  |  |  |  |  |  |  |
| PbF3H | LOC103953484 | 11.8792 | 13.0252 | 13.8942 |  | 16.8372 | 17.0525 | 15.1955 |
|  |  |  |  |  |  |  |  |  |
| Flavonol synthase | |  |  |  |  |  |  |  |
| PbFLS | LOC103933697 | 4.17832 | 6.52509 | 5.35786 |  | 1.06924 | 1.76584 | 1.14147 |
|  | LOC103956721 | 0.190711 | 0.0001 | 0.086081 |  | 0.0001 | 0.0001 | 0.0001 |
|  |  |  |  |  |  |  |  |  |
| UDP-glycose flavonoid 3-O-glycosyltransferase | | | |  |  |  |  |  |
| PbUFGT | LOC103951514 | 8.80153 | 8.2945 | 7.85304 |  | 3.58637 | 4.07272 | 3.6891 |
|  |  |  |  |  |  |  |  |  |
| Quercetin O-methyltransferase | |  |  |  |  |  |  |  |
| PbQMOTb | LOC103943352 | 193.568 | 193.989 | 193.867 |  | 202.99 | 202.842 | 191.046 |
| - | LOC103951568 | 0.1208 | 0.0001 | 0.0001 |  | 0.117144 | 0.08739 | 0.08161 |
| - | LOC103951569 | 0.104026 | 0.089207 | 0.281592 |  | 0.302679 | 0.302636 | 0.353434 |
| PbQOMTa | LOC103951578 | 1.45341 | 1.54839 | 0.760957 |  | 0.586566 | 0.964816 | 0.573989 |

**Table S2** Primers and gene accessions

|  | Accession | Gene name | Forward primer (5'-3') | Reverse primer (5'-3') |
| --- | --- | --- | --- | --- |
| qPCR | LOC103967156 | *PbMYB12a* | TTTATCCCACCCTGCTGGAC | CTCTCGACCGAGCCAAGAAT |
|  | LOC103931986 | *PbMYB12b* | TTGATGGAGGGTTGTTGGGG | GATGATGATCCGCGTCCGTA |
|  | LOC103965055 | *PbCHSa* | ATGGTTACCGTCGAGGAAGT | CGGGATATGTGGCTTGATCC |
|  | LOC103965056 | *PbCHSb* | TAGCGTGGGTTTAACGGCTT | CCTTACATGCACGGTTCCCT |
|  | LOC103933697 | *PbFLS* | GCCGGTGGTGATAACTTGGA | TGAACATCGTTGGGGACGAG |
|  | LOC103951514 | *PbUFGT* | TCGAAGAACTCGACCTCCCT | ATGGAGCGTCTTGCTTGTCA |
|  | LOC103926850 | *PbActin* | CCATCCAGGCTGTTCTCTC | GCAAGGTCCAGACGAAGG |
|  |  |  |  |  |
| CDS | LOC103931986 | *PbMYB12b* | ATGGGGAGGGCGCCGTGCTGT | ATGGGGAGGGCGCCGTGCTGT |
| RNAi | LOC103931986 | *PbMYB12b* | ATAGGCAGCAAGCATTTGGT | GGTAGGGAACTGGTGCTGAT |
| Promoter | LOC103933697 | *PbFLS* | GAATGTATAAAGTTCGCTAGATTCAA | TGGTCACACTCCCTTTACGTGAAG |
|  | LOC103965056 | *PbCHSb* | TTATAATCGCTTAGCTACTCTTCTC | TGAATAAGAAATATGGGGCAGAAATG |
